# Characterization of an Advanced Blast Simulator for Investigation of Large Scale Blast Traumatic Brain Injury Studies

**DOI:** 10.1007/s10439-024-03618-6

**Published:** 2024-09-14

**Authors:** Allison J. Nelson, David Ritzel, Noah Showalter, Danny Boppe, Andy Riegel, Pamela J. VandeVord

**Affiliations:** 1 School of Biomedical Engineering and Sciences, Virginia Tech, Blacksburg, VA USA; 2 Department of Biomedical Engineering and Mechanics, Virginia Tech, Blacksburg, VA USA; 3 Dyn-FX Consulting Ltd, Amherstburg, ON Canada; 4 Stumptown Research and Development LLC, Black Mountain, NC USA; 5 Veterans Affairs Medical Center, Salem, VA USA

**Keywords:** Advanced blast simulator, Primary blast injury, Traumatic brain injury (TBI), Preclinical model, Blast wave

## Abstract

Blast traumatic brain injury (bTBI) is a prominent military health concern. The pervasiveness and long-term impacts of this injury highlight the need for investigation of the physiological outcomes of bTBI. Preclinical models allow for the evaluation of behavioral and neuropathological sequelae associated with bTBI. Studies have implemented rodent models to investigate bTBI due to the relative small size and low cost; however, a large animal model with similar neuroanatomical structure to humans is essential for clinical translation. Small blast simulators are used to induce bTBI in rodents, but a large animal model demands a larger device. This study describes a large advanced blast simulator (ABS4) that is a gas-detonation-driven system consisting of 5 sections totaling 40 ft in length with a cross-section of 4 × 4 ft at the test section. It is highly suitable for large animals and human surrogate investigations. This work characterized the ABS4 in preparation of large-scale bTBI testing. An array of tests were conducted with target overpressures in the test section ranging from 10 to 50 psi, and the pressure-time profiles clearly illustrate the essential characteristics of a free-field blast wave, specifically a sharp peak pressure and a defined negative phase. Multiple blast tests conducted at the same target pressure produced very similar pressure profiles, exhibiting the reproducibility of the ABS4 system. With its extensive range of pressures and substantial size, the ABS4 will permit military-relevant translational blast testing.

## Introduction

### Blast Traumatic Brain Injury

Blast traumatic brain injury (bTBI) is the predominant type of head injury in combat military populations. More than 490,000 military service members have sustained a traumatic brain injury (TBI) since the year 2000 [1]. Operation Enduring Freedom and Operation Iraqi Freedom resulted in a greater proportion of head and neck injuries compared to previous conflicts, and blast exposure was found to account for 78% of these injuries during combat [2].

Because of advancements in medical technology and body armor, the survivability rate for recent conflicts is higher than that of prior wars; however, ‘invisible wounds’, including impairments from TBI due to blast exposure, emerged as new clinical challenges in combat service members [3, 4]. An array of symptoms is associated with bTBI, including headaches, dizziness, difficulty with balance, fatigue, alterations in sleep habits, memory and attention deficits, and behavioral and mood changes [3], but unlike penetrating injuries, these health conditions are often invisible to the eye and, in many cases, go unacknowledged [4]. Current treatments of TBI focus on minimizing secondary injury and providing rehabilitation services, but no pharmacological therapies or effective treatments for full functional recovery exist [5, 6]. The prevalence and lack of efficacious treatment emphasize the importance of understanding the physiological effects of bTBI.

**Fig. 1:**
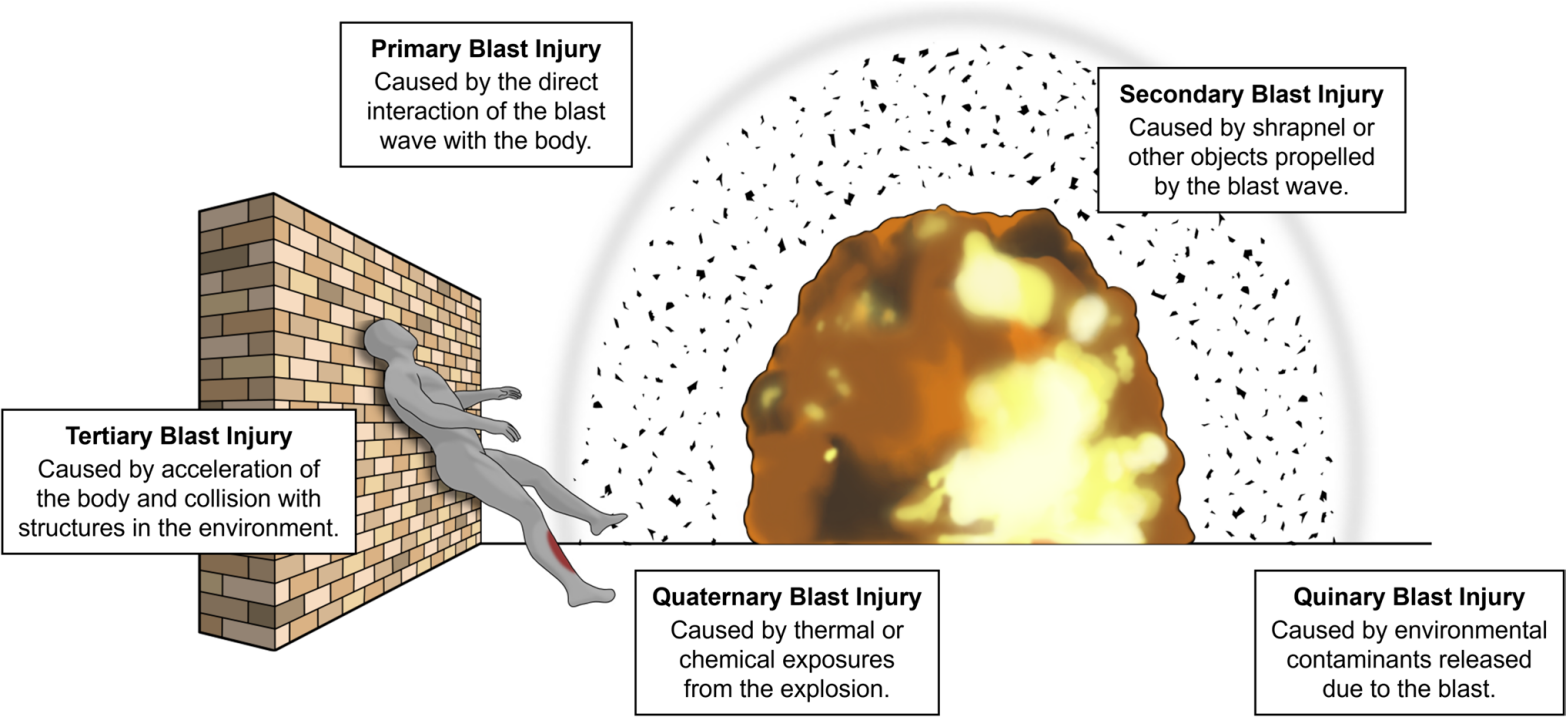
Illustration of blast injury categories outlined in DoD Directive 6025.21E

Injuries due to blast are classified into five categories based on the Department of Defense (DoD) Directive 6025.21E and are outlined in Fig. 1 [7].

Briefly, primary blast injury is damage caused by the interaction of the blast wave with biological tissue. Secondary blast injury is caused by shrapnel or other objects propelled by the blast wave. Tertiary blast injury is caused by the acceleration of the body due to the blast wind and collision with structures in the environment. Quaternary blast injury is caused by thermal or chemical exposures from the explosion, typically occurring in close proximity to the explosion source. Lastly, quinary blast injury is caused by environmental contaminants that were released due to the blast, such as bacteria or radiation. The mechanism of primary bTBI has been highly speculated upon and several theories have been proposed [8]; however it remains elusive.

Military-relevant preclinical models of bTBI are essential for investigating the unique mechanisms of injury, understanding subsequent sequelae and behavioral changes, identifying relevant biomarkers and injury thresholds for diagnosis and prognosis, and testing effective treatment strategies [9]. Rodent models of TBI continue to be valuable for prefatory investigations due to their small size, cost-effectiveness, and well-established collection of physiological and behavioral data; however, the inclusion of a large animal model as an intermediate species between rodents and humans would greatly improve clinical translation [10]. Small animal species such as the rodent have a lissencephalic neuroanatomical structure, meaning the rodent brain lacks cortical gyri and sulci that are present in the gyrencephalic human brain and may affect the brain’s biomechanical response [11, 12]. In addition, gyrencephalic brains typically have a higher subcortical white-to-gray matter ratio than lissencephalic brains [11], and this distribution is important to consider in translational model selection as injury vulnerability is different in white and gray matter structures [13]. Furthermore, developing an injury threshold for humans using preclinical data requires scaling from an animal to a human. In terms of brain mass, the rat brain would have to be scaled by a factor of more than 800, while a larger animal model, such as the minipig, would only need to be scaled by a factor of about 20 [14]. Large animals with gyrencephalic brains, such as pigs or sheep, also have a more similar brain mass to skull thickness ratio to humans than rodents do [15]. Skull thickness is another aspect suspected to play a role in the biomechanical response of the brain during blast and should also be considered when scaling bTBI models [8, 16]. Beyond the factor of mass, scale also affects the hydrodynamics of the blast interaction. For the same blast exposure conditions, small specimens the scale of rats are disproportionately affected by ‘blast throw’ forces and global acceleration rather than the transient crushing action of the hydrostatic pressure which dominates for objects the scale of a human head [17].

Small blast simulation systems with cross-section widths ranging from 9 to 24 inches have been used for bTBI rodent studies [18–20], but a larger animal model requires a larger blast simulator to allow for appropriate blast wave physics to develop within the test section [21, 22]. Norris et al. studied the effect of low clearance around a surrogate human head on the blast waves in a comparison between an advanced blast simulator (ABS) with a 1 ft cross-section width and the large 4 ft cross-section width ABS presented in this publication [23]. In a free-field blast exposure, the blast wave will completely engulf a specimen, and the static and dynamic pressure interacting with the specimen geometry causes diffraction and a ‘wrap-around’ blast loading effect. In order to not impede this ‘wrap-around’ loading of the blast flow, the specimen should not exceed more than 20% of the cross-sectional area of the blast simulator [9, 24].

The large advanced blast simulator (ABS4) located at the Virginia Tech (VT) Center for Injury Biomechanics is the first of its kind to be used for larger animal and human surrogate bTBI studies. It is imperative that an experimental blast model adequately replicates a free-field blast in a laboratory setting for meaningful results and translation [9]. Therefore, the objective of this work was to assess the performance of the ABS4 and evaluate its ability to replicate the characteristics of a free-field blast prior to the introduction of an animal or human surrogate model.

### Blast Physics

**Fig. 2:**
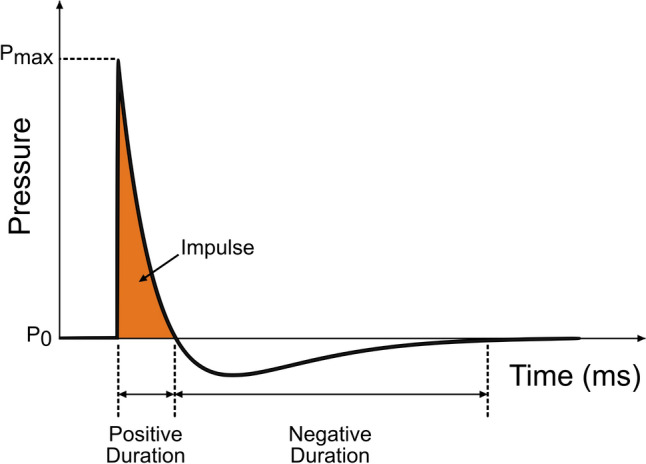
Plot illustrating the idealized Friedlander waveform and identifying defining characteristics of a blast wave

It is crucial that when designing an experiment to investigate blast injury, the fundamental physics of blast are well understood to avoid deceptive results and a misunderstanding of the injury and associated risk [9]. When an explosion is detonated, a ‘fireball’ is formed as the combustion gases expand at high velocities, and the leading edge of the expanding fireball drives the shock front into the surrounding atmosphere [9, 25]. The near-field region includes the zone engulfed by the expansion of the fireball and consists of complex flow conditions including both shocked air and detonation products. The mid-field region is beyond the expansion of the fireball, but the flow conditions are still nonuniform [8]. Only in the far-field regime does the blast wave exhibit a uniform quasi-one-dimensional flow where waveforms can be approximated by the idealized Friedlander equation, characterized by a near-instantaneous rise from ambient pressure to peak overpressure, a rapid exponential decrease in overpressure, a period of negative pressure, and a return to ambient pressure (Fig. 2). Although sometimes misunderstood, the Friedlander equation is simply an empirical curve-fit approximation of an actual blast waveform. Actual spherical or cylindrical blasts will feature a secondary shock for example, usually early in the negative phase, which is typically 10–15% of the primary shock amplitude. Although effective for approximating the positive phase, neither the secondary shock nor the true character of the negative phase are properly considered in the Friedlander fit.

Several quantitative parameters are used to describe a free-field blast wave, such as peak static pressure, duration, impulse, and wave speed. Peak static pressure is the maximum pressure level during the near-instantaneous rise due to the shock front. The positive duration is the time from the onset of the shock front to when the pressure falls below ambient conditions. The impulse is the integral (area under the curve) of the pressure-time profile during the positive phase. Wave speed can be assessed using the arrival time of the blast at two distances from the blast source, and this information can be used to validate other measured parameters, such as peak pressure, using Rankine–Hugoniot relations [26, 27].

**Fig. 3:**
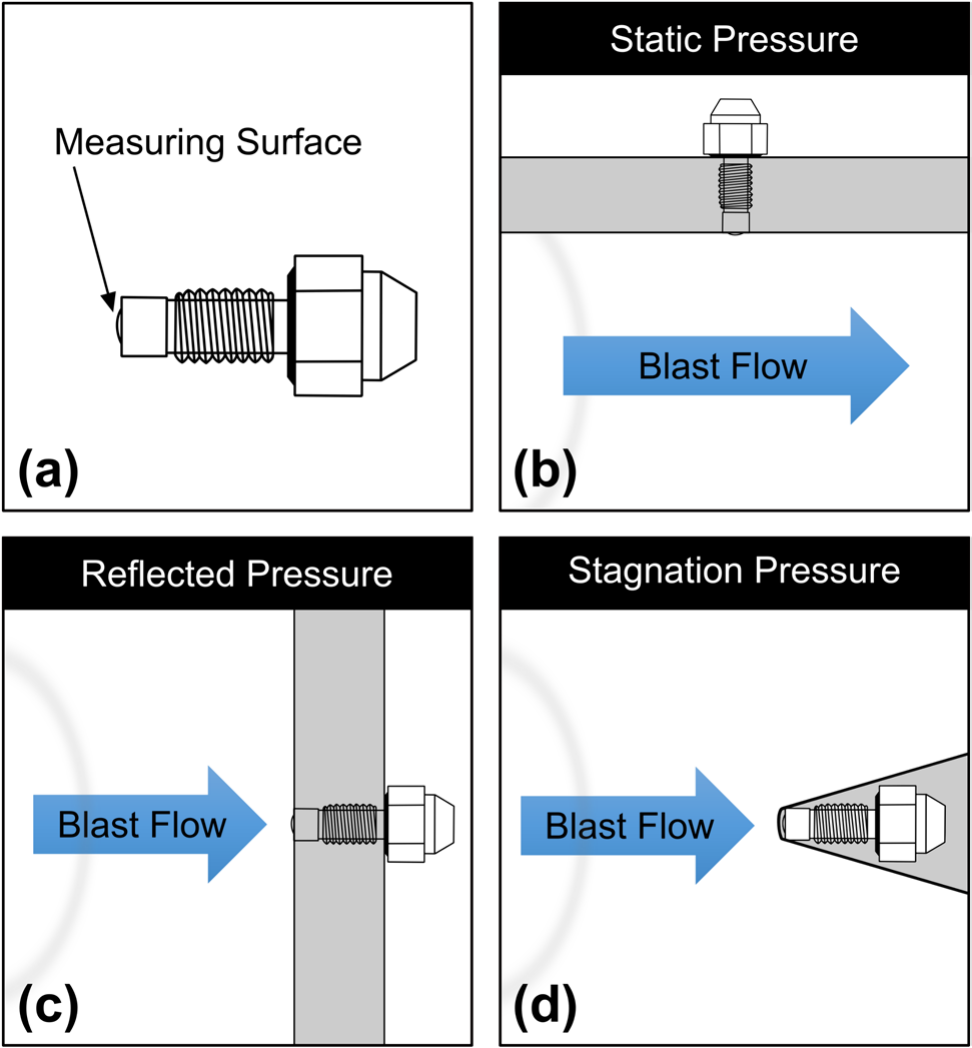
Illustration of how different pressures are measured in a blast setting **a** Identifies the measuring surface of an Endevco Model 8530 pressure sensor, for example **b** Shows the pressure sensor measuring surface is parallel to, and does not impede, the blast flow **c** Shows the reflected pressure measured perpendicular to the blast flow. Note that the reflected pressure is a function of the blast strength and the angle of incidence **d** Shows stagnation pressure is measured perpendicular to the blast flow with a specially shaped pencil probe

When discussing pressure pertaining to a blast wave, it is important to define which type of pressure is being described. Technically, ‘static pressure’ is the thermodynamic state of uniform principal stress for a gas, that is, the pressure created due to compression or heating; this condition is independent of any consideration of the kinetic energy of the flow, and must be measured perpendicular to the flow of the blast wave as in the side-port of a Pitot-static gauge, often known as a ‘pencil’ probe [24]. Static pressure is the primary factor for transient global crushing action on an object. Dynamic pressure is a measure of the kinetic energy of the blast flow, and while it cannot be directly measured, with certain assumptions, it can be calculated by subtracting the static pressure from the stagnation, or total, pressure [9, 28]. This total pressure is the sum of the static and dynamic pressures and is measured in practice using the tip port of a pencil probe in a quasi-steady flow where the flow is brought to rest [24, 28]. The dynamic pressure is the primary factor for ‘blast throw’ forces, and its relative intensity changes with blast strength. For example, for a 10 psi static pressure blast, the dynamic pressure is slightly greater than 2 psi, whereas for a 100 psi static pressure blast, it exceeds 120 psi [29, 30]. Reflected pressure is the pressure caused by the reflection of the shock wave off a rigid surface; although similar conceptually to stagnation pressure in that the flow is being brought to rest, the process is different through ‘non-isentropic’ shock dynamics. Measuring reflected pressure allows for the characterization of the load a surface is experiencing in a blast [25], which can be of particular interest in understanding blast injury mechanisms or designing protective equipment to prevent injury. The magnitude of reflected pressure is determined by the intensity of the incident blast wave and the angle at which the blast wave strikes the surface [28]. Interestingly, the maximum reflected pressure in most cases of practical interest (5–50 psi) is not at normal reflection, but at an intermediate angle between 40° and 55° due to the phenomena of Mach reflection [24, 28]. Figure 3 illustrates how each of these pressures can be measured in a blast scenario.

### Producing a Blast Wave

Explosive field tests, while valuable for some validation purposes, are costly and highly inefficient for systematic blast research. Additionally, apart from the expense, access to the specialized field test sites and personnel is increasingly difficult. Although open-field detonations may seem to replicate realistic combat scenarios, inherent variability between explosive events and lack of control of environmental conditions does not allow for the consistency and repeatability required for statistical significance in research [31]. The logistics of animal care and application of advanced diagnostic methods are extremely challenging in a field environment. Furthermore, with open-field blasts, secondary injuries are likely to be caused by fragmentation and ejecta, so additional preventative measures must be used to ensure injuries are caused only by the blast wave.

Therefore, devices have been developed to replicate explosive blast conditions for the laboratory environment. The conventional compressed-gas shock tube is one of the most popular devices for laboratory blast simulation. In its simplest form, a shock tube consists of two constant-area cylindrical pipe sections with a frangible membrane separating a high-pressure ‘driver’ section from the low-pressure ‘driven’ section; the membrane is ruptured by a mechanical device or simple over-pressurization to propagate a shock wave into the driven section. A conventional compressed-gas shock tube can create a shock wave with a decaying profile but can only simulate blast-wave exposure conditions to a limited extent and only if the test article is carefully positioned [9, 24]. The shock wave profile can approximate the positive phase of an explosive blast at a substantial distance from the frangible membrane.

In open-end shock tubes, the shock wave is reflected as it reaches the open end and a rarefaction wave is generated which propagates back upstream greatly accelerating the outflow and causing a collimated high-speed jet to form external to the tube. The upstream rarefaction causes an overexpansion of the flow resulting in a following upstream recompression shock. Therefore, if a specimen is subjected to the open-end rarefaction wave, it will be exposed not only to the initial shock wave but an exaggerated surge of outflow velocity followed by a recompression shock striking from the back. This problem highlights the need for an ‘end-wave eliminator’ device such that no adverse wave effects are propagated back to the test section where experiments are staged [9].

The ABS, patented in 2011 [32], is a specially contoured shock tube designed to replicate the key characteristics of blast wave flow conditions, including the negative phase and secondary shock, as well as eliminate artifacts seen with conventional shock tubes attempting blast simulation [9, 32]. A key feature of the ABS is the inclusion of an end-wave eliminator (EWE) device at the end of the simulator. The EWE consists of a shock diffuser within a dump tank; the shock diffuser breaks up the shock front, counteracting the rarefaction wave, and the dump tank prevents the expanding gases and noise from venting into the lab space [9]. The ABS driver has a specially contoured divergent shape which expands the flow geometrically causing development of the negative phase and a secondary shock. The subsequent transition section gradually re-converges the flow without incurring anomalous transverse waves before the wave enters the test section. These alterations to the conventional shock tube allow for the development of a shock wave profile that accurately replicates the characteristics seen in a far-field explosive blast.

## Materials and Methods

### VT ABS4 Description

**Fig. 4:**
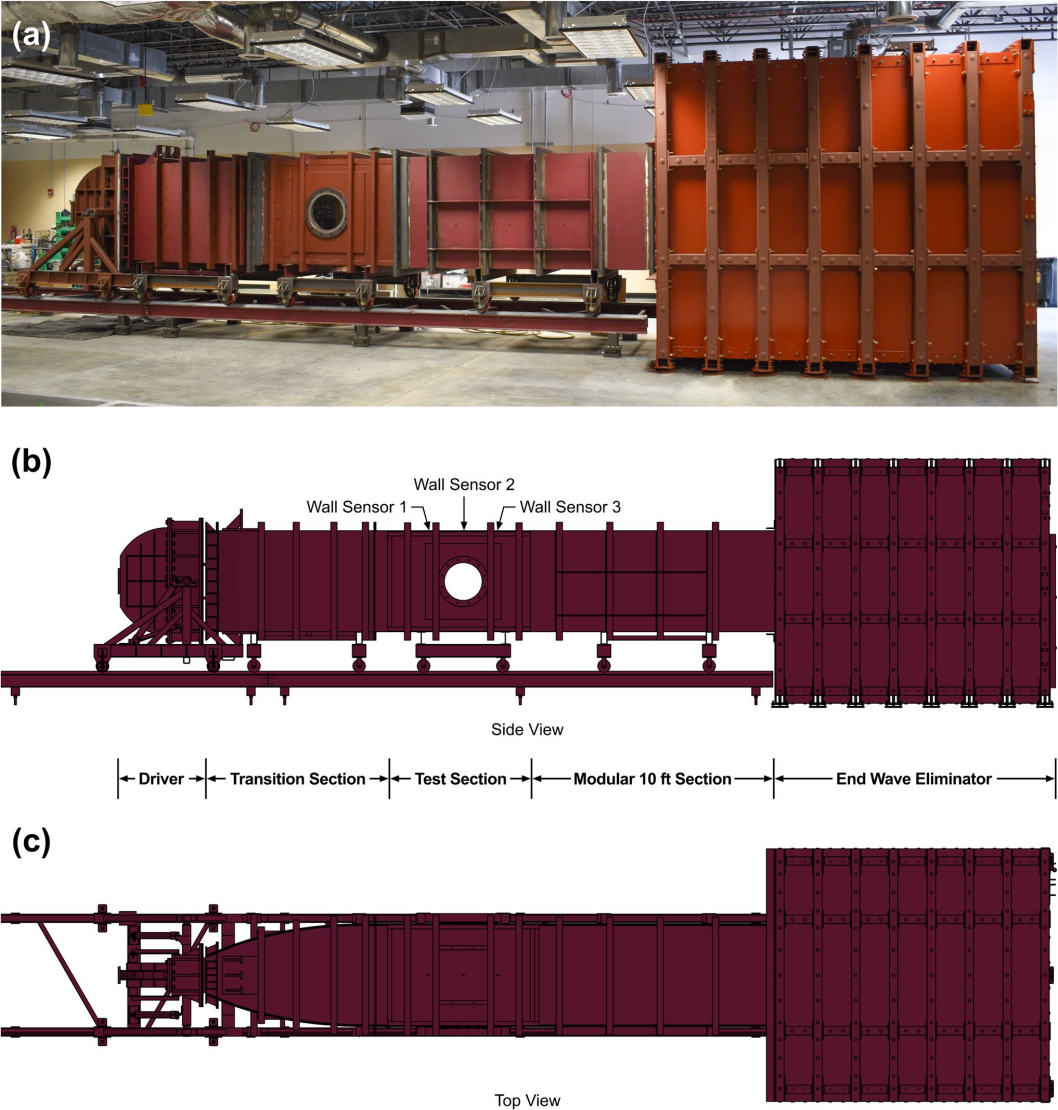
**a** Image of the ABS4 located at the Virginia Tech Center for Injury Biomechanics and drawings of the **b** side view and **c** top view of the ABS4

The VT ABS4 (Stumptown Research and Development LLC, Black Mountain, NC) is 40 feet long, constructed of steel, and comprised of five separate sections (Fig. 4a, b). The divergent driver has a wedge-shape that leads into the expanding transition section (Fig. 4c). The test section, where specimens are typically placed for blast exposure, is 6 feet long with a cross-section of 4 ft by 4 ft. It has 16-inch diameter acrylic windows on each side to allow for high-speed video capture and observation of live specimens. In the current study, the modular 10 ft (in length) section was located between the test section and the EWE as shown in Fig. 4, but it can be moved closer to the driver to increase the stand-off distance of the test section. Since the ABS was designed to produce a fully-formed blast wave beyond the transition section, specimens can be placed anywhere between the transition and the EWE, unlike in a conventional shock tube. The shock diffuser of the EWE contains an array of structural steel angles that can be reconfigured in order to provide the appropriate blockage ratio to reduce unwanted end effects. The blockage ratio can be increased to counteract overexpansion of the blast wave or decreased to counteract reflection of the blast wave back upstream in the simulator. The dump tank, which houses the EWE shock diffuser, was designed to contain the expanding gases and mitigate sound for safety. The driver, transition section, test section, and modular 10 ft section are mounted on wheels and can move along a raised track that joins against the stationary EWE.

The ABS4 is a gas detonation system that uses a mixture of oxygen and acetylene to generate an elevated pressure region when ignited with an electric match. The gases are supplied to the driver section of the ABS4, and a thin membrane is used to contain the gas mixture within the driver section until detonation. During testing, the ABS4 gas supply system is run with a computer and a control panel located in the lab space adjacent to the ABS room. The gas flow controllers (MCR-Series, Alicat Scientific, Tucson, AZ) are operated with FlowVision 2.0 Software (Alicat Scientific, Tucson, AZ). Gas is supplied to the driver in two phases. In the first phase, oxygen gas is released into the driver to flush the air out of the contained volume, establishing an oxygen rich environment conducive to acetylene detonation. In the second phase, oxygen and acetylene are injected into the contained volume at the stoichiometric ratio, 5 parts oxygen to 2 parts acetylene by volume. Once the ABS4 driver is filled, the control panel is used to light the electric match and ignite the oxy-acetylene mixture. All lab personnel move to the adjoining lab space and don hearing protection prior to filling the driver with the oxy-acetylene mixture.

The peak pressure of the blast wave is varied by adjusting the ratio of oxygen to acetylene, the total gas volume, and the driver volume containing the gas mixture. While the physical dimensions of the driver are fixed, the volume that contains the gas mixture can be adjusted by moving the location of the thin membrane.

### Instrumentation

**Fig. 5:**
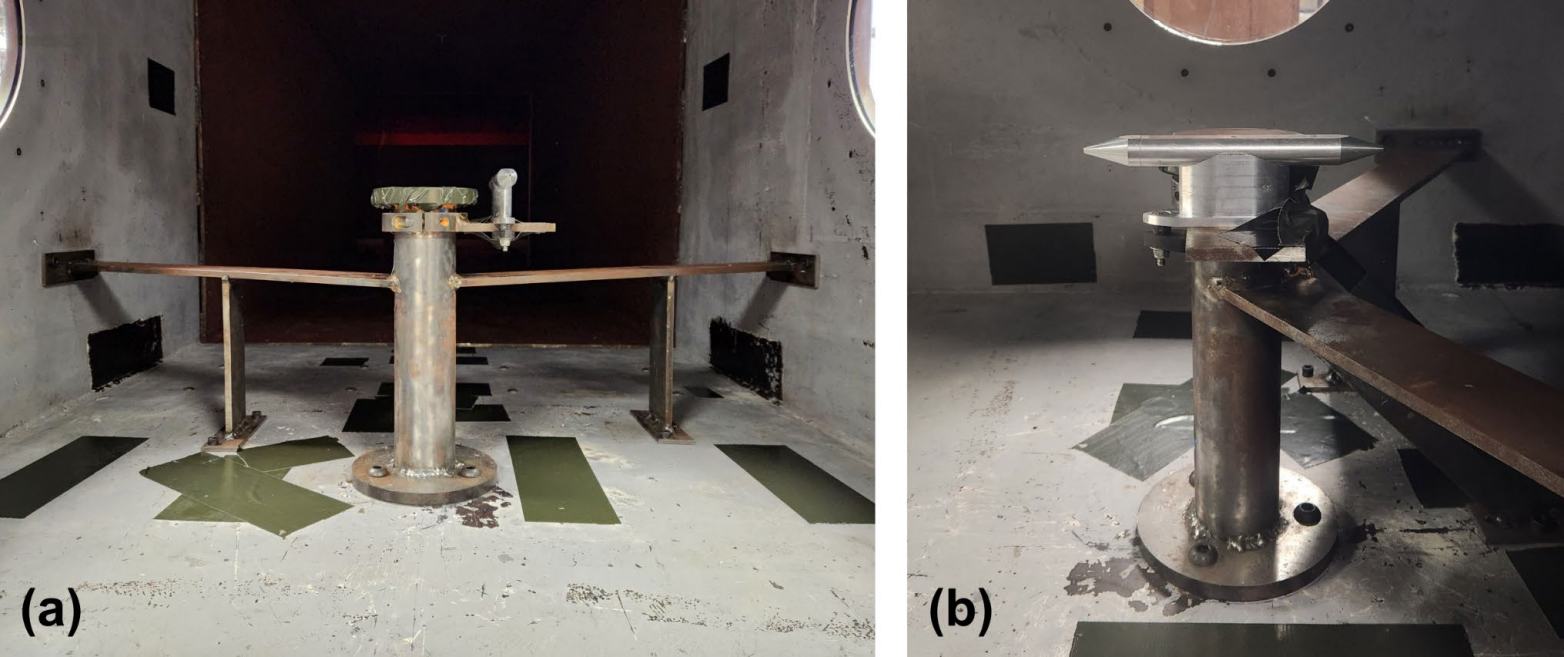
Images of the pencil probe secured to a post in the center of the ABS4 test section from a **a** frontal view and **b** side view

For the characterization tests, three pressure transducers (Model 8540-200, Endevco, Depew, NY) were mounted flush with the wall in the top of the test section of the ABS4 to measure static overpressure. These three sensors were located 149.5 inches, 165 inches, and 180.5 inches from the back of the driver, with the Wall Sensor 2 located in the middle of the test section. All three transducers were secured in threaded nylon fixtures within the wall of the ABS4 to reduce acceleration and electrical noise. The cables for the transducers were secured outside of the ABS4 on foam to dampen vibration and noise. A two-transducer (Models 8530B-500 and 8530C-100, Endevco, Depew, NY) aluminum pencil probe (Stumptown Research and Development LLC, Black Mountain, NC) was placed on a post in the center of the test section (Fig. 5) and measured stagnation pressure and static overpressure. All data were conditioned and acquired using a TMX Multi-Channel High Speed Data Acquisition Recorder (AstroNova Inc., West Warwick, RI), and signals were sampled at 800 kHz.

In this characterization study, five blast tests were completed at each target pressure of 10, 20, 30, and 50 psi. Multiple blasts were also conducted at a target pressure of 12 psi to evaluate the consistency of the ABS4. Blast parameters including peak static pressure, impulse, duration, and wave speed were calculated from the collected data for each target pressure. Peak static pressure, impulse, and duration are reported from Wall Sensor 2, which is located in the wall in the middle of the test section. Wave speed was computed using the time between the arrival of the shock front as measured by Wall Sensors 1 and 3 and the distance between the two sensors. Peak dynamic pressure was computed for 3 blast tests using the pencil probe stagnation and static pressure sensors.

## Results

**Fig. 6:**
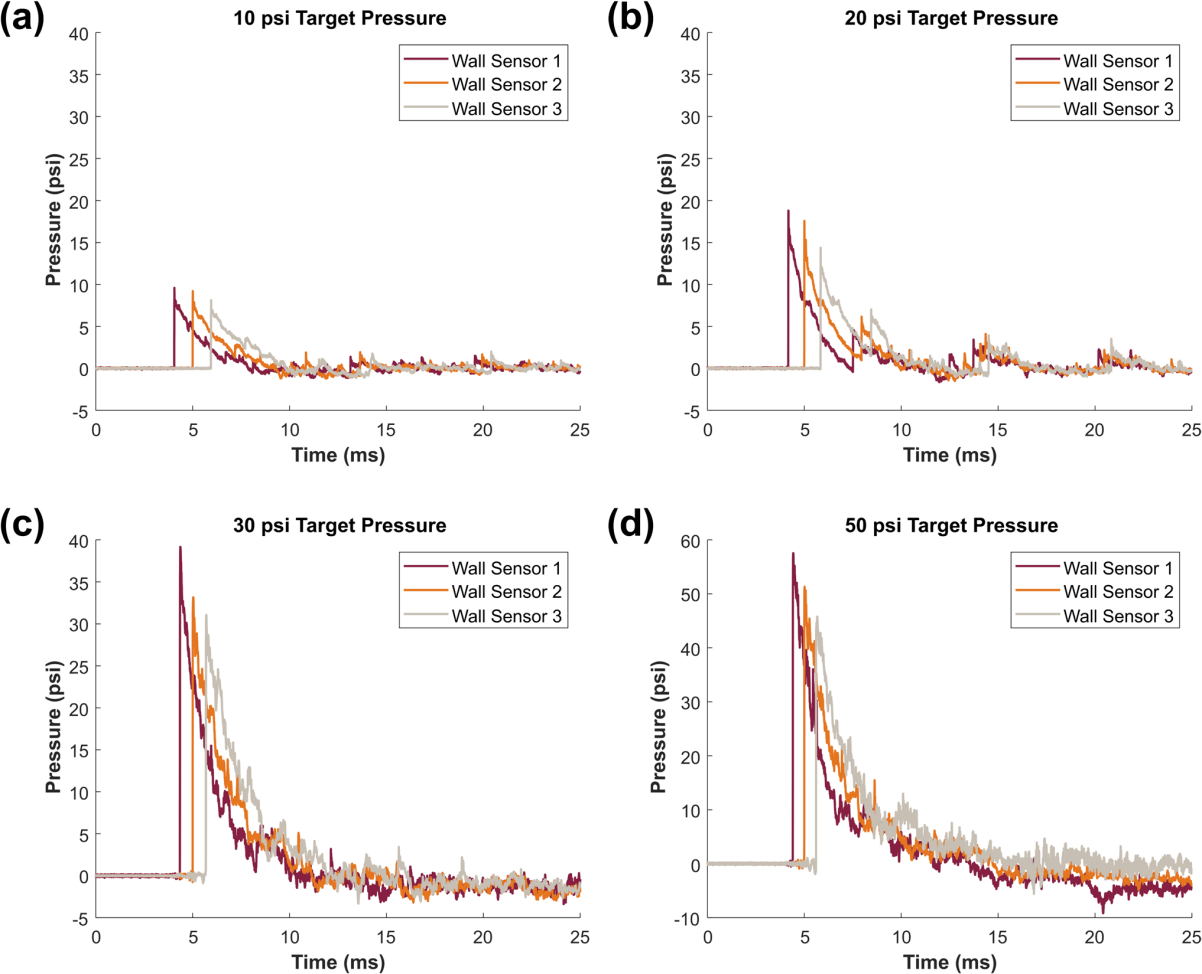
Representative static pressure plots for pressures of approximately **a** 10 psi, **b** 20 psi, **c** 30 psi, and **d** 50 psi measured by the three wall sensors located in the test section of the ABS4. The reported pressure level was determined by Wall Sensor 2 as it is mounted in the wall in the middle of the test section above where a specimen would likely be located

Representative static overpressure traces from the three wall sensors in the test section of the ABS4 for pressures of approximately 10, 20, 30, and 50 psi are presented in Fig. 6. Based on the Wall Sensor 2 pressure trace, the positive phase begins at 5 ms for each pressure level in the representative plots, and the negative phase begins in the representative plots for 10, 20, 30, and 50 psi at 9.2, 10.1, 11.2, and 14.5 ms, respectively. Parameters including peak pressure, duration, impulse, and wave speed were determined for five blast tests at each target pressure (*n* = 5), and the averages ± standard error are presented in Table 1. Dynamic pressure was computed as the difference between the stagnation and static pressures collected with the pencil probe. The peak dynamic pressure was determined for 3 blast tests at each target pressure (*n* = 3), and the averages ± standard error are presented in Table 1. As the peak static pressure increased, dynamic pressure, duration, impulse, and wave speed all increased as well.

**Table 1:** Blast wave parameters determined for tests at approximately 10, 20, 30, and 50 psi

Peak static pressure (psi) *n* = 5	Peak dynamic pressure (psi) *n* = 3	Positive phase duration (ms) *n* = 5	Impulse (psi-ms) *n* = 5	Wave speed (m/s) *n* = 5
9.76 ± 0.33	2.71 ± 0.14	4.45 ± 0.07	11.87 ± 0.65	421.62 ± 2.12
18.75 ± 0.35	5.40 ± 0.74	5.70 ± 1.49	27.24 ± 0.19	483.31 ± 3.41
30.40 ± 0.97	10.69 ± 1.17	6.09 ± 0.24	50.22 ± 2.12	556.84 ± 9.45
49.85 ± 1.22	30.48 ± 2.91	12.54 ± 0.96	122.26 ± 7.01	675.63 ± 9.08

**Fig. 7:**
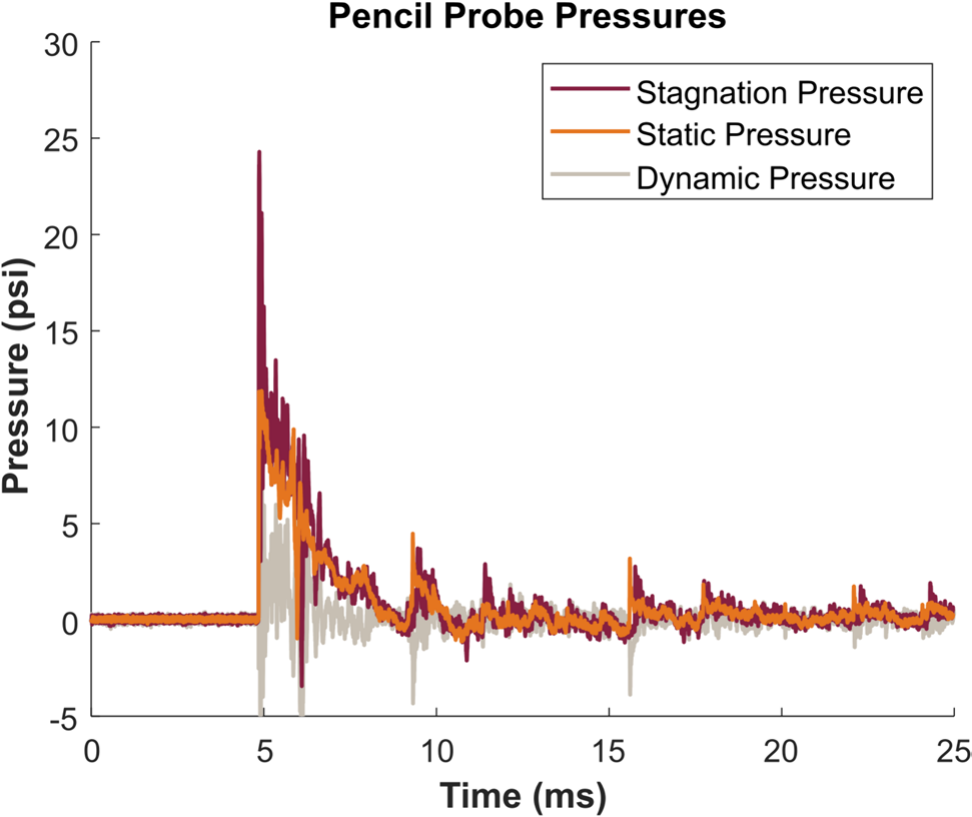
Representative pressure-time plots of stagnation, static, and dynamic pressures measured with the pencil probe

Fig. 7 shows a plot of the stagnation pressure and static pressure measured with the pencil probe and dynamic pressure, which was computed as the difference between the stagnation and static pressure. The target for this test was 12 psi static pressure. Note that the dynamic pressure component is relatively low compared to the static pressure, as is expected for a blast of this strength in the far-field regime [24]. The peak dynamic pressure values reported in Table 1 are comparable to theoretical peak dynamic pressure values for each peak static pressure level [30].

**Fig. 8:**
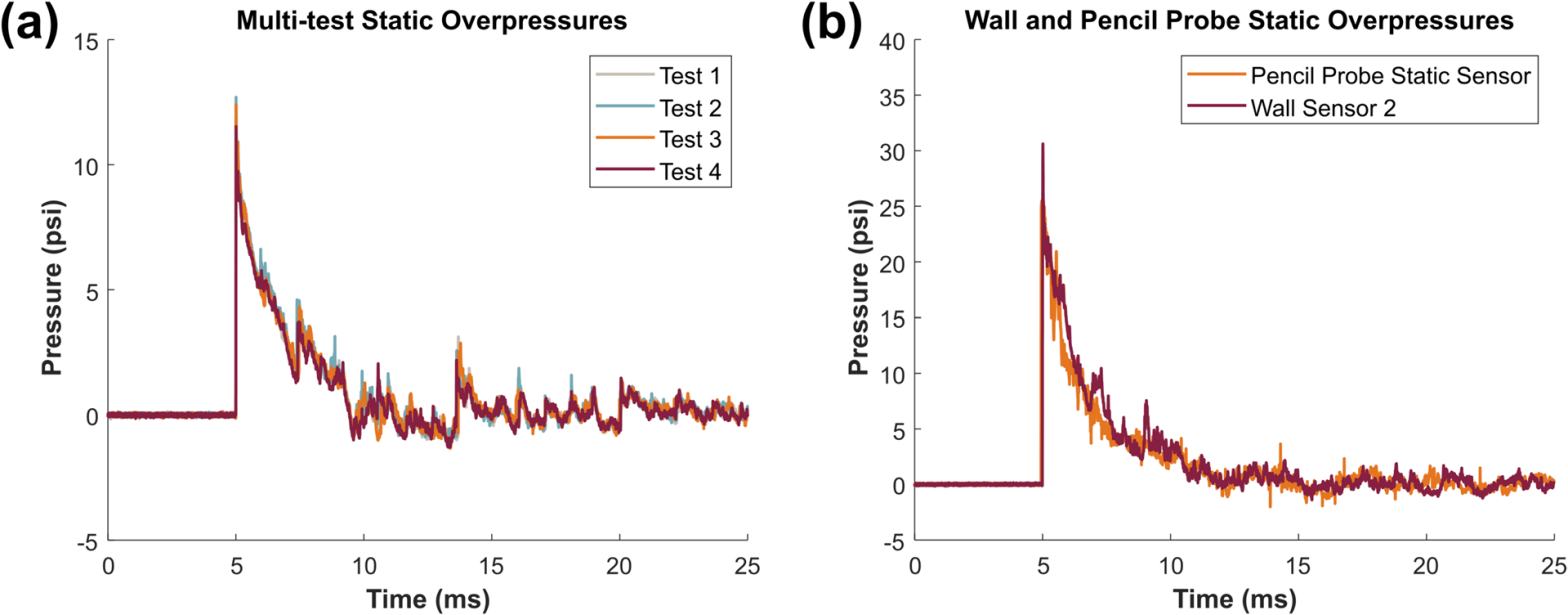
**a** Pressure-time plots overlaid from multiple tests at the same target static pressure (12 psi) to highlight repeatability of the ABS4 and **b** Pressure-time plots at a target static pressure of 30 psi measured at the edge of the ABS4 with a wall sensor and at the center of the ABS4 with the pencil probe static sensor

The repeatability of the system was evaluated by overlaying the static pressure traces for multiple trials at the same target pressure of approximately 12 psi (Fig. 8a). The multiple tests were arbitrarily aligned in the time domain by the onset of the blast wave. Note that a secondary shock appears in the record about 9 ms after the primary shock, somewhat after the start of the negative phase as seen in open-field spherical blast. Planarity of the blast wave was evaluated by comparing static pressure traces from the sensor located in the wall in the middle of the test section, Wall Sensor 2, and the pencil probe static pressure sensor located in the center of the test section (Fig. 8b). The blast wave not only arrives at the same time, but the decay of the blast wave is also similar in the center and at the edge of the ABS4 test section.

## Discussion

In the described study, pressure measurements taken across multiple trials in the test section of the ABS4 signify the ability of the ABS4 to recreate the defining characteristics of a blast wave and the exceptional repeatability of the system. The ABS4 demonstrated its ability to produce an expansive range of pressures, extending from around 10 to 50 psi. Furthermore, while only this range was highlighted in this publication, the ABS4 system is not strictly limited to these pressures. Other preliminary testing with this system has indicated that it is currently capable of producing static overpressures with blast profiles resembling those seen in the far-field region of real-world blasts as low as 3 psi and as high as 70 psi. This broad range capability of the ABS4 will permit clinically-relevant blast levels for single and repeated exposure studies. Military service members are routinely exposed to blast overpressure during training exercises and combat scenarios through breaching activities and operation of heavy weapons [33]. Some service members have reported headaches, memory impairments, sleep-wake disturbances, and ringing in the ears after repeatedly performing these activities that fall within the safe incident pressure threshold of 4 psi established by the U.S. Army doctrine [34]. The effect of this cumulative low-level blast exposure has not been widely investigated, and the use of translational preclinical models with the ABS4 could help elucidate the effects of these subconcussive injuries and establish safe stand-off distances and safety thresholds for not only incident pressure levels but also for frequency of exposure. High-level blast, typically defined as incident pressure levels greater than 8 psi for injury studies, is also a continuing concern. These pressure levels often result in primary bTBI ranging from mild to severe [35]; however, there remains a paucity of information on the mechanism responsible for these injuries. The ABS4 is able to produce a wide range of high-level blast pressures to investigate the mechanism of injury for primary bTBI and perhaps establish dose-response relationships for different severities of bTBI. Moreover, while protective equipment such as combat helmets has been shown to provide protection against ballistic and blunt trauma, it was not designed to mitigate neurotrauma from primary blast [36]. The ABS4 along with preclinical or human surrogate models of bTBI could be used to evaluate the effectiveness of current military protective equipment in both low-level and high-level blast environments and delve into possible improvements for protection against blast neurotrauma.

Future work will consist of customizing the fuel mixtures and evaluating stoichiometric, lean, and rich oxy-acetylene mixtures to most efficiently achieve target pressures. Furthermore, the scope of performance for the ABS4 will be explored to expand the pressure-impulse range. It would be valuable to have the ability to adjust the duration and negative phase using louvered venting as demonstrated in Gan et al. [37] without altering the peak static pressure and maintaining the hallmark characteristics of real-world blasts. Ultimately, the ABS4 allows for the capability of large animal and human surrogate models of blast injury. The considerable size of the ABS4, the comprehensive range of pressure levels the system is able to produce, and the repeatability of the system will allow for expansive translational and military-relevant blast injury studies.
